# “Sport can unite people, but not with them, they don't love this country” ethnic prejudice and identity among basketball fans in North Macedonia

**DOI:** 10.3389/fspor.2025.1617447

**Published:** 2025-10-31

**Authors:** Arin Agich, Luca Váradi, Tamás Dóczi

**Affiliations:** ^1^ Doctoral School, Hungarian University of Sports Science, Budapest, Hungary; ^2^ Nationalism Studies Program, Central European University, Vienna, Austria; ^3^ Department of Social Sciences, Hungarian University of Sports Science, Budapest, Hungary

**Keywords:** intergroup prejudice, social identity theory, North Macedonia, contact hypothesis, post-conflict society

## Abstract

This study explores how ethnic identity and intergroup prejudice are shaped, expressed and challenged within basketball fandom in North Macedonia, a multi-ethnic and divided society. While sports fan culture is widely recognized as a platform where group belonging and identity are developed, there is limited qualitative research in the Balkans that examines how these identities intersect with ethnic divisions in everyday fan practices. In particular, this study looks at whether sports can serve as a tool for inclusion and reducing ethnic-based prejudice among fans in post-conflict and multi-ethnic societies. To address this gap, we conducted six focus group discussions with 30 members of ethnic-Macedonian and ethnic-Albanian basketball fan groups. Using thematic analysis, we analyzed (a) how fan identities are shaped by group and ethnic belonging and expressed through group symbols, loyalty, and rituals; (b) how intergroup prejudice and exclusion are expressed through perceptions of rivalry and national representation, and (c) whether extended intergroup contact can reduce prejudice among fans. Our findings reveal that fan identities are intertwined with broader socio-political narratives, and that sporting spaces often reinforce, rather than bridge, symbolic boundaries. In addition, Extended Contact Hypothesis (ECH) remains largely ineffective due to emotional detachment and conditional acceptance of the other*.* These insights offer further understanding of the role of sports and the limitations of contact-based interventions in divided societies, such as North Macedonia.

## 1 Introduction

“It is the reason I wake up every morning”, answered one of the fans from North Macedonia without hesitation and with passion when asked why he supports his team. This reply sets the stage for exploring the compelling relationship between fandom and its intimate connection to identity, belonging, and the perception of the other within sports.

**Figure 1:**
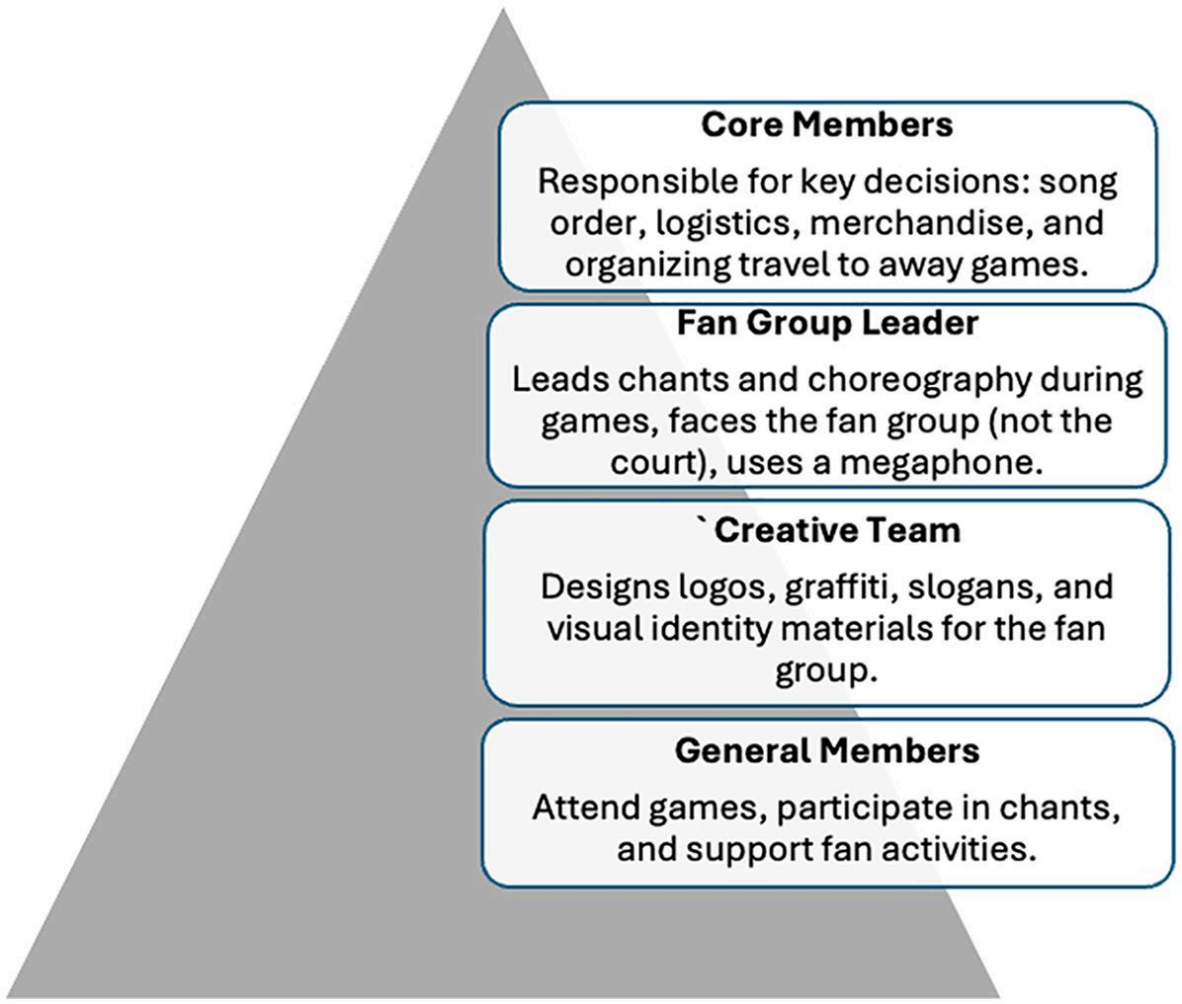
Fan group structure.

**Figure 2:**
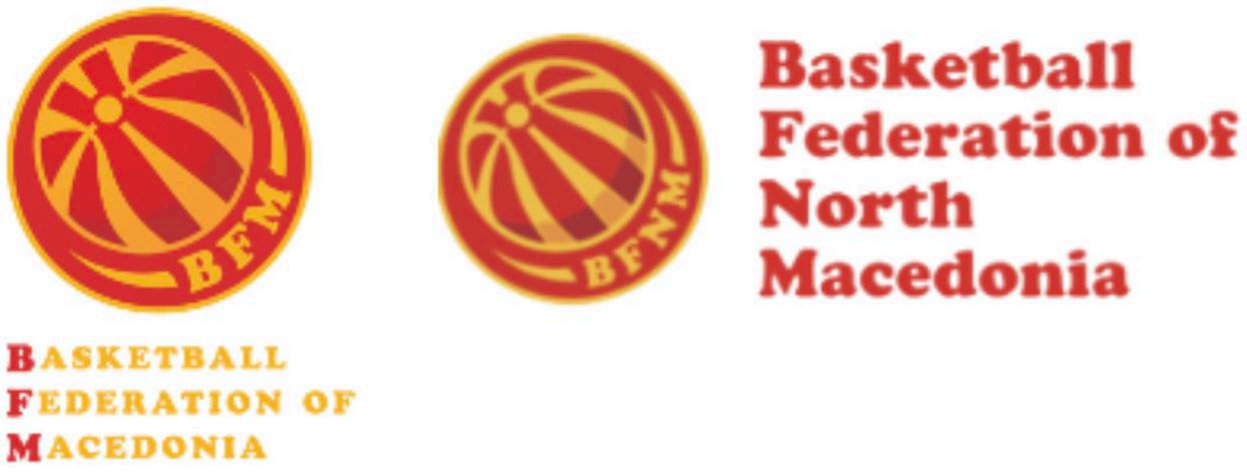
Logo of the basketball federation before and after the change of the country's name (google images, 2025).

**Table 1:** Focus groups composition.

Focus group (FG)	Ethnic affiliation	Club supported	Age range	Number of participants	Fan group affiliations
FG1	Macedonian	Team 1	25–45	5	Group leader + core supporters
FG2	Macedonian	Team 2	25–30	5	Core supporters
FG3	Macedonian	Team 3	20–35	5	Core supporters
FG4	Albanian	Team 4	20–30	5	Group leader + core supporters
FG5	Albanian	Team 5	20–30	5	Group leader + core supporters
FG6	Albanian	Team 6	20–30	5	Group leader + core supporters

Fandom has caught the attention of social scientists and has become a vital subject of research due to its impact on identity construction, social belonging, collective behavior, its relationship to intergroup bias, and eventually violence (1–4).

In the present study, we focus on how ethnic identities and intergroup prejudices are expressed, reinforced, and challenged within the context of basketball fan culture in North Macedonia. Exploring the inter-ethnic relations in the Balkans has been undertaken from various perspectives, yet, the phenomenon has scarcely been explored in the realm of sports, nor has it been researched using qualitative methods. In this paper, we present a thematic analysis of six focus groups (total of *N* = 30 fans) uncovering how fan identities are shaped by ethnic belonging, how intergroup prejudice is expressed through rivalry, and whether Extended Contact Hypothesis (ECH) within sports can lead to prejudice reduction in divided societies. The focus groups were conducted with members of the country's largest and most influential fan groups, who at the same time are members of the ethnic-Albanian (minority) or ethnic-Macedonian (majority) communities.

Sports fans are known to establish identities through their support and attachment towards specific sport teams or individual athletes by creating cohesive communities marked by strong emotional attachment, self-identification, and collective social behaviors (5, 6). In addition, fandom has been seen to profoundly impact individuals' self-concept and identity by providing significant meaning and adding structure in their lives (7, 8). For instance, they often describe their support towards their chosen teams as a core value of their identity and existence, frequently using pronouns such as “we” when referring to their team, illustrating a type of merging of individual and group identities (4, 5). According to Jacobson (8), sports fan identity development typically progresses through stages including initial attraction, increased involvement, and eventual identity internalization, where the fan's self-concept becomes intertwined with their chosen sports team or athlete. Jacobson (8) also emphasizes that this identification process not only provides personal fulfillment and social belonging but can also result in intensified perceptions of rivalry, which can further reinforce group cohesion and boundary creation against opposing groups. Individuals' self-identification has been seen to go beyond and integrate towards broader socio-cultural narratives, influencing their behavior within sporting context and beyond. In line with this, as Antonsich (9) argues, belonging emerges both from personal identification with a group and from the sense of being accepted and recognized by others. In addition, Yuval-Davis's (10) “politics of belonging” argues how belonging is shaped by power relations and socio-political dynamics, especially in contested contexts. With that said, in the realm of sports fandom, this duality is highly relevant. Identification with a team may signal emotional attachment and solidarity, but it also intersects with broader political and ethnic dynamics, especially in societies marked by division such as in the case of North Macedonia.

Another important concept of group identification and its role in identity formation can be described through Social Identity Theory (SIT). Initially formulated by Tajfel and Turner (9), this framework offers a refined understanding of fan behavior, particularly in relation to how individual identities are shaped through group membership. According to SIT, individuals derive a significant portion of their self-concept and self-worth from the groups they belong to, leading them to favor their “in-group” and distance themselves from perceived “out-groups” (7, 9). In the context of fandom, this approach helps to examine how loyalty is expressed, how symbolic boundaries are drawn, and how belonging is affirmed through emotional investment and rituals. Rather than treating group attachment as neutral, SIT brings attention to the ways in which collective identity is performed, defended, and experienced, especially in polarized or high-stakes environments. Lock and Funk (4), in their study on sport fandom, highlight the role of social identity and its formation through team affiliation, which can strongly influence emotions, behaviors, and attitudes, even extending to aggressive or prejudiced behavior towards rival fan groups.

In addition, symbols, clothing and color play a vital role in social identity development among fans, serving as tangible markers that strengthen group boundaries by reinforcing intergroup distinctions. The concept of “enclothed cognition” explains how clothing influences psychological processes. Adam and Galinsky (11) introduced this term to describe the systematic impact that clothes have on the wearer's cognitive functions. Their research suggests that the symbolic meaning attributed to certain clothing can affect the wearer's behavior and self-perception. In the context of traditional and hot forms of sports fandom, wearing team jerseys or colors showcase specific meanings and it can influence behaviors aligned with the group's norms. According to Giulianotti (12), “supporters” are known for their intensity, with long-term emotional investment and local affiliation, mainly expressed through visible and embodied practices such as various clothing pieces (e.g., jerseys with their team's logo).

According to SIT, the processes of in-group favoritism and out-group differentiation simultaneously create the ground for intergroup tensions, competition and prejudice. Prejudice among sports fans manifests as negative attitudes, stereotypical perceptions, and discriminatory behaviors, directed towards out-group members, in this case, the rival team's fans and players.

This understanding aligns with Allport's (13) definition of prejudice as an “antipathy based on faulty and inflexible generalizations” which, in the case of fans, may manifest in actions ranging from derogatory slogans, verbal slurs to outright aggression during (and after) sporting events (12). This can especially be seen in multi-ethnic and multi-religious societies, where sport fan communities frequently reflect and mirror broader societal tensions in the sporting venues, allowing existing ethnic, religious, political, cultural and other divisions to become highly visible (1). To further understand the reasoning of such prejudice in divided societies, Realistic Conflict Theory (RCT), which was initially developed by Sherif (14) and later expanded by Jackson (15), explains that intergroup prejudice arises when groups perceive themselves to be in direct competition over limited resources. These can be materialistic, such as territory and access, or symbolic, such as status, legitimacy and cultural dominance (16). In the context of fan culture, this perspective offers a way to understand how groups come to see others as threats not only to physical space or institutional power, but to identity and recognition itself. RCT helps to explore how perceptions of zero-sum rivalry are constructed and maintained, especially when shaped by political histories, spatial segregation, or unequal representation. This makes it particularly relevant for exploring settings where sport becomes a vehicle for expressing deeper tensions over who belongs, who leads, and who is seen as legitimate. To further elaborate on the nature of rivalry in sports, Tyler and Cobbs (17) argue that rivalries are not purely based on competition but are deeply rooted in fans' perceptions of cultural significance, history, and identity. Their research shows that certain competitions are perceived as more intense due to factors such as geographic proximity, historical conflict, and cultural differences (17). With that said, in post-conflict, divided societies these factors make sports rivalries a potent platform for expressing broader social tensions.

In addition to social tensions, and to better capture the emotional dynamics revealed among the fans, we also draw on affect theory (18), which highlights how emotions are not only personal but also shaped by social and political contexts. In sports fandom, these emotions circulate within groups, attach to symbols such as teams, flags, or songs, and influence how fans draw boundaries between “us” and “them.” Feelings such as pride, resentment, and detachment are not just reactions they are part of how group identities are formed, maintained, and sometimes challenged. From this perspective, expressions of emotional detachment and conditional inclusion are not signs of neutrality but rather affective strategies that fans use to manage contradiction, uncertainty, and social pressure. Fandom, then, becomes a space where emotions and identities are deeply entangled where prejudice is also felt, managed, and at times, resisted.

Fan identities and the way they express their support towards their team frequently intersects with political ideologies, social movements, ethnic or religious affiliations, impacting fans' attitudes toward rival groups (1, 2). For instance, one of the famous fandom rivalries in Scotland is between the football teams Celtic and Rangers, rooted in religious and political tensions between Catholics and Protestants. Matches between these teams often feature chanting and violence reflecting the religious and political division within the Scottish society (19). Another prominent football example includes the ethnically charged, infamous rivalry between the Croatian team Dinamo Zagreb and Red Star Belgrade from Serbia which, in 1990 during the dissolution of Yugoslavia, ended in fan violence indicating how sports rivalries can embody broader political and ethnic conflicts (15).

Just like many other sports, basketball also serves as an arena for the expression of ethnic identities and intergroup prejudice, especially in multi-ethnic and multi-religious societies such as the ex-Yugoslav countries. For example, the fierce basketball rivalry between Partizan and Crvena Zvezda from Serbia, MZT-Skopje and Cair 2030 from North Macedonia, and the basketball teams of Kosovo such as Peja and Prishtina extend beyond mere athletic competition, reflecting the lasting impact of nationalism and division in post-Yugoslav societies (17). These examples from various sports collectively illustrate how fandom is deeply interwoven with ethnic, national, religious and political identities. Therefore, sport acts as a critical social platform where intergroup relations, including prejudice and conflict, are prominently displayed and at times potentially mitigated through shared sportsmanship (5).

Speaking of reducing prejudice with the aim to curb potential aggression and violence among sports fans, Wright and his colleagues' (20) ECH shows that knowing an in-group member who has a positive relationship with an out-group member can reduce one's prejudice toward that out-group. In the context of fans, this theory suggests that fans observing or being aware of positive relationships between players of different ethnic and social backgrounds can lead to more positive perceptions and attitudes among the fans themselves. For instance, sociologists Gomez and Huici (21) conducted a study with 107 basketball fans in Spain to explore the role of ECH on prejudice reduction. The fans watched a video showing a positive joint training session between their own team and a rival team, where the results showed that this form of extended contact improved fans' general attitudes toward the rival team. These effects were stronger when the interaction was approved by the coach, suggesting that both social endorsement and indirect exposure to positive intergroup relations can play a vital role in diminishing prejudice (20).

Beyond academic interest, the role of sport as a tool for fostering inclusion, social cohesion, and peace has also been recognized, promoted and widely communicated by federations, international organizations and sporting institutions. For instance, The United Nations recognizes the role of sport in contributing to the Sustainable Development Goals (SDGs), particularly promoting education, gender equality, and peaceful societies (22, 23). Similarly, the International Olympic Committee (IOC) asserts that sport is a cost-effective tool to promote peaceful coexistence and human development (22).

Furthermore, there are initiatives at the European level, such as the EPAS program of the Council of Europe, and strategies sponsored by the EU that underscore the role of sport in transcending ethnic, cultural, and social divides, also in post-conflict reconciliation and integration (52, 53). As highlighted in previous research (22, 23), an array of programs like Open Fun Football Schools in the Balkans and FARE Action Week throughout Europe demonstrate how intentionally designed sports programs have the potential to unite fractured communities. While these initiatives have clear objectives, the processes and results tend to differ greatly depending on the local context, the institutional level and commitment. While these initiatives have clear objectives and goals, the outcome of programs may vary depending on the local context and the commitment of the institutions. There are certain empirical studies which provide a deeper understanding of their effectiveness. For example, a randomized controlled trial by Salma Mousa (24), which examined whether a mixed football league could help social cohesion among displaced Christians in post-ISIS Iraq. The results showed that Christians assigned to mixed teams with Muslim players were more likely to engage in tolerant behaviors within the sporting context, such as voting for Muslim peers to win a sportsmanship award or registering for future mixed teams (24). However, outside of the sporting context, the intervention had limited impact. These findings highlight that while well-designed sports programs can foster inter-group tolerance, their effects may not easily be generalized without broader social support (52).

The possibility of using sport to reduce intergroup prejudice is often explored through contact-based interventions (CBI) as the ones mentioned earlier, which are designed to facilitate positive interaction between members of different social, ethnic, or religious groups. These interventions are based on the Contact Hypothesis and its later developments, such as the ECH, and aim to create structured environments where participants can experience cooperation, equal status, and shared goals. In the context of sport, CBI includes mixed teams, collaborative tournaments, joint fan initiatives, and other forms of meaningful engagement. While some studies have shown promising results in reducing bias and fostering tolerance, outcomes often depend heavily on the social and emotional context in which contact occurs. In this study, we do not examine only the potential but also the limitations of such interventions in a deeply divided and emotionally charged society like North Macedonia.

It is within this context that North Macedonia presents a compelling case. In multi-ethnic and multi-religious societies like North Macedonia, fan culture can be perceived as a valuable lens to examine how identities and prejudices are articulated reflecting the broader societal tensions and historical division within the country. In addition, it also gives the possibility to explore whether prejudice and negative attitudes can be reduced through sports, in a deeply divided and post-conflict context, particularly in settings where direct contact between different ethnic groups has been limited.

After gaining independence in 1991, North Macedonia, with a total population of 1.8 million, became home to diverse ethnic and religious groups. The 2021 census revealed that ethnic Macedonians now constitute 58.44% of the population, followed by ethnic Albanians at 24.30%, and smaller communities including Turks, Roma, Bosniaks, Vlachs, and Serbs comprising 6.35% collectively (25). The primary religious groups include Orthodox Christians (46.14%) and Muslims (32.17%), which contribute significantly to the cultural and linguistic diversity of the country (25). This diversity, however, has been accompanied by persistent ethnic tensions rooted in historical grievances, particularly following the dissolution of Yugoslavia. The emergence of nationalist ideologies among newly independent Balkan states contributed to increased interethnic tensions, nearly escalating into a civil war in 2001 between ethnic-Albanian militants and Macedonian security forces (26, 27). The Ohrid Framework Agreement (OFA), mediated by international actors including the EU and NATO, ended the conflict by providing greater autonomy and political representation to ethnic Albanians (28). Nevertheless, tensions persist, exemplified by recent controversies surrounding the official use of the Albanian language and incidents of nationalist provocations during Albanian Flag Day celebrations in 2024, highlighting ongoing sensitivities and divisions (28).

A significant development shaping ethnic and national identities in North Macedonia was the country's official name change from the “Republic of Macedonia” to the “Republic of North Macedonia” in 2019 (28). This decision followed decades of diplomatic disputes with Greece, which had strongly opposed the use of the name “Macedonia” without a geographical indicator, citing concerns about territorial claims and historical appropriation (29). After prolonged negotiations and significant domestic political controversy, the Prespa Agreement was signed in 2018 between North Macedonia and Greece, leading to constitutional amendments and the official adoption of the new name in 2019 (30). This decision was met with polarized reactions domestically where many ethnic-Macedonians perceived it as an erosion of national identity and sovereignty, resulting in widespread protests and political instability (29, 30). On the other hand, supporters viewed the name change as essential for the country's prospects of NATO membership and European Union integration, arguing that resolving the dispute was necessary for long-term geopolitical stability and regional reconciliation (30). This issue, reflecting deeply rooted questions of identity and nationhood, inevitably influenced social dynamics, including sports culture, where national symbols and identities are frequently contested and reaffirmed (1).

Sports in North Macedonia, particularly handball, football and basketball, have frequently served as microcosms of these broader ethnic tensions. Incidents involving violence, nationalist chants, and confrontations among fans, such as those between ethnic-Macedonian supported teams (e.g., Vardar) and ethnic Albanian-supported teams (e.g., Shkëndija), underscore how sport events become focal points for the expression of ethnic identities and rivalries (31). Despite the ideal of sports as a unifying force, in North Macedonia, sports events often reflect and intensify existing societal divides, highlighting persistent challenges in interethnic integration and coexistence.

In addition, it is important to highlight the role of urban segregation when it comes to reinforcing further division among different ethnic groups within society, especially in the capital city Skopje. Unlike more ethnically homogenous cities like Tetova and Struga (predominantly with ethnic-Albanians), Skopje's diverse population is split along ethnic (and religious) lines, with the river Vardar being both a symbolic and physical marker of the division. The northern side of the city predominantly hosts ethnic-Albanians and other minorities, while ethnic Macedonians primarily occupy neighborhoods on the southern side (25). Historically shaped by urban planning decisions from the Ottoman era, through Yugoslavian governance, and intensified following independence in 1991, this division has fostered an environment of limited interethnic interaction, significantly affecting the social perceptions and daily lives of its inhabitants (32, 33). This division is apparent on a daily basis, with many ethnic minorities attending schools which teach in their own language, hang out in cafés/bars/restaurants owned by people coming from the same ethnic groups, and, finally, attending homogeneous sport clubs with owners, coaches, players, and fans from the same community (34). This urban segregation extends deeply into sports culture, where it is manifested not only through ethnically homogeneous teams and fans but also in the physical locations and naming of sports facilities. Famous sporting venues such as Boris Trajkovski Sports Center and Jane Sandanski Sports Center, located in predominantly Macedonian southern municipalities, contrast sharply with venues situated in the predominantly Albanian northern neighborhoods like Cair Sports Center and Shaban Tërstena Sports Hall, reflecting clear ethnic affiliations through the choice of the individuals after whom the venues were named (34). In addition, this urban division is further reinforced through symbolic markers, such as national flags, monuments of historical figures, and religious symbols displayed prominently in different neighborhoods.

This segregation also affects fan interactions and athlete compositions within the teams, reinforcing ethnic prejudices and divisions rather than providing opportunities for meaningful cross-ethnic engagement and contact. The most popular sports in North Macedonia are football, handball, and basketball (35, 36). Though football is considered North Macedonia's most developed and successful sport, in the last decade basketball has also drawn the attention of many sport lovers. After the success of North Macedonia's men's National Basketball Team in the European Championship hosted by Lithuania in 2011, where the team finished in fourth place out of twenty-five European countries for the first time in history (37), basketball as a sport spread among the country's population like never before.

The current basketball teams within the Basketball Federation of North Macedonia are categorized according to age and gender (35). Although there are no rules that mark ethnic division among the teams, the ones located in ethnically homogenous cities and neighborhoods (25) have a roster of non-Macedonian players (identifiable by their surnames and/or names). Similarly, teams identified as Macedonian primarily consist of ethnic-Macedonian players. It is important to note that in accordance with North Macedonia's Law on Free Access to Public Information (38), which ensures transparency by allowing individuals and legal entities to access public information held by state authorities and other organizations, a formal request was submitted to the Basketball Federation of North Macedonia. This request sought demographic data on players and teams within the federation for scientific research purposes. Despite the legal obligation to respond, the federation failed to provide the requested information.

A typical basketball team includes a management team (or club owner), a coach, assistant coach, twelve players, and, in a broader sense, the fans. While not formally listed on the roster, fans are increasingly recognized in sport sociology as integral members of a team's extended identity, contributing emotionally, symbolically, and performatively to its dynamics and meaning (2, 6, 12). Depending on the league level (professional, semi-professional, or amateur) and the team's popularity and success, teams receive varying degrees of financial and emotional support, often manifested through sponsorships and the presence of dedicated fan groups.

Fan groups for teams competing in lower leagues (second, third or fourth division) are usually smaller and more informal, consisting of friends, family members, and basketball enthusiasts who enjoy supporting the sport regardless of the competitive level also known as social fans (39). However, fan groups associated with professional teams function as structured, interdependent organizations integral to the team's identity (39). The primary purpose is unwavering support shown through consistent game attendance throughout the entire basketball season, writing and composing songs and slogans, printing promotional materials such as scarfs, t-shirts, and flags, and organizing special events aimed at rallying support or celebrating certain team achievements (e.g., winning the game against the biggest rival, winning the championship, or ranking on higher positions in the league table). Importantly, the influence of fan groups over the teams which they support often extends beyond mere support. It also significantly impacts the strategic decisions of team management, including hiring or firing team staff (coaches) and players (40).

Although research on sports fandom exists on a large scale for the international sports scene, studies on fan behavior in North Macedonia remain limited and largely focused on isolated aspects of fan dynamics rather than in-depth exploration on matters such as ethnic identity and prejudice. For example, Anastasovski et al. (41) explore whether family relationships are significant when measuring deviant fan behaviors during sports events in North Macedonia. Their quantitative approach uncovers sociological risk factors but does not address broader identity issues or interethnic relations. Similarly, research conducted by Ameti and his colleagues (42), explored the organizational structure, historical development, and fan satisfaction of FC Shkëndija from Tetovo, offering insights into club management but without exploring deeper ethnic and identity related dimensions of fan behavior. In addition, Petreski and his colleagues (43) analyzed sports fan groups as a platform for promoting right-wing extremist ideologies in North Macedonia, Serbia and France, focusing specifically on ideological indoctrination rather than on everyday ethnic interactions and identity formation among fans (51, 53). Ibraimi (44) in his study highlights the role of sports-related hate speech as political propaganda in the former Yugoslav region, including North Macedonia, but primarily addressed broader nationalist narratives without examining fan perspectives or employing qualitative methodologies to capture their voices directly. Moreover, although media coverage and investigative journalism in North Macedonia frequently report on fan related violence, nationalist incidents, and problematic behavior at sports events (45, 46), they do not explore the subjective experiences, identities, or interethnic perceptions of the fans themselves. Consequently, there remains a significant gap in the scholarly understanding of how (basketball) fans in North Macedonia construct, express, and negotiate ethnic identity and prejudice through their interactions and collective behaviors.

Through focus groups and directly engaging with members of North Macedonia's largest fan groups, this study aims to address the existing gap by bringing insights into how ethnic identities and intergroup dynamics may be expressed or challenged within basketball fandom. To address these aims, our study is guided by the following research questions:

1. How do basketball fans in North Macedonia express their identities within the context of ethnic belonging and fan culture?
2. Do fan interactions reflect and reinforce ethnic prejudice or inter-ethnic understanding?; How are these attitudes and rivalries expressed?
3. Does extended contact in sports reduce prejudice among basketball fans in a divided, multiethnic society?

## 2 Materials and methods

To explore how ethnic identity, social identity, intergroup dynamics and prejudice are expressed and experienced among basketball fans in North Macedonia, we conducted a qualitative study. Qualitative methods are especially suited for exploring how individuals make sense of their social realities, particularly in contexts marked by societal divisions, tensions, and boundaries (1).

In the field of sports studies, qualitative approaches have proven effective in capturing the nuanced ways in which fans construct and negotiate group affiliation, identities, rivalries and the perception of the out-group (1, 3). This approach also opens the floor to go beyond survey-based research in the field of fandom by engaging more directly with fans' practices and lived experiences.

More specifically, this study employs focus group discussions as the primary data collection method. Focus groups are especially effective for researching attitudes and beliefs in collective settings, as they may uncover shared social norms, tensions, perceptions, and group narratives (47, 48). Given that the fan culture is both an individual and collective practice shaped by interactional dynamics, focus groups provide an excellent environment for examining how ethnic Macedonian and ethnic Albanian basketball fans engage with and construct narratives about belonging, interethnic relations, and political influence in sport.

The data gathered through focus groups were analyzed using reflexive thematic analysis, a method which allows the identification of patterns of meaning across qualitative datasets (49). Thematic analysis (TA) is well-suited for capturing both manifest content (what is explicitly said) and latent meaning (underlying themes and ideologies), making it appropriate for exploring complex issues. In this study, while the focus group questions and prompts were derived from prior literature on social identity theory (19), prejudice (13), and Extended Contact Hypothesis (20), the analysis itself was conducted inductively, allowing themes to emerge from the data.

### 2.1 Recruitment and sampling

Participants of the study were recruited through already established networks within the field of basketball in North Macedonia, specifically in Skopje. These were basketball coaches, former and current basketball players, and local political figures active in the field of sport, such as supporting local clubs, organizing events, or shaping sports policy, with whom there was already existing contact. Recruitment was purposive, with the aim to include active fans of the most prominent basketball clubs and rivalries in North Macedonia, with a special focus on ensuring balanced representation across ethnic groups. Key figures such as fan group leaders were initially contacted and invited to participate. Each leader was then asked to bring four additional fans, resulting in focus groups of five participants each. In cases where further clarification was needed, especially due to fear of exposure and past negative experiences among fans participating in similar group discussions, the first author communicated directly with the potential participants by phone to further clarify regarding the discussion. Participants were also given an information sheet which included a short description of the study, confirmation of anonymity and data protection, and contact details of the moderator of the focus groups (the first author of this paper).

In total, six focus groups (see Table 1) were conducted in Skopje, North Macedonia, in January, 2025: three with ethnic Albanian fans and three with ethnic Macedonian fans, resulting in a total of 30 fans (*N* = 30). Each group included five fans. Participants' ages ranged from 20 to 45 years, with an estimated mean age of 27.5 years. All members were long-term and currently active supporters of their respective basketball teams. All participants were male, as no female fans responded, even though there was no indication in the invitation regarding gender.^1^

Participants in this study were active members of four of the largest and most influential basketball fan groups in North Macedonia, which typically range in size from 300 to 2,000 members. The size and stability of these groups often depend on their historical legacy, organizational structure, successful recruitment of new generations, and financial capacity to sustain engagement, merchandise and group activities. According to the focus group discussions, each fan group is organized hierarchically, with a distinct subgroup of “core” members who are responsible for key logistical and strategic decisions. These include the composition, selection and order of songs and chants, the design and distribution of merchandise, and the coordination of trips to away games. In addition, many groups have a “creative” team tasked with producing visual materials such as banners, logos, slogans, and graffiti, which serve as expressions of group identity and territorial occupation within their respective neighborhoods. At the top of this structure is the group “leader,” a central figure who directs in-game choreography, initiates chanting, and maintains the group's energy and coordination. The leader typically stands facing the fan section, rather than the court, using a megaphone to communicate with the group. The leader is chosen according to the “sacrifices” done in the name of the group, including active membership throughout many years. All the fan groups also include sub-groups with distinct names, often based on the specific areas of their neighborhoods where their members come from. This structure has been shown to support faster mobilization and wider outreach, particularly when recruiting new members. The hierarchical way of organization plays a significant role in shaping how group norms are established, reinforced, and publicly displayed during games.

All focus groups included the presence of a group leader, often the person responsible for coordinating chants, logistics, or internal group communication. As such, they held a degree of symbolic authority within the fan group. Their presence inevitably shaped the dynamics of the discussions, as other participants occasionally sought non-verbal approval before speaking or echoed dominant viewpoints. The moderator was attentive to these patterns and actively worked to balance participation, encouraging quieter voices to contribute and posing follow-up questions when group conformity appeared to restrict expression. While the group setting allowed for capturing shared norms and narratives, it also highlighted how power hierarchies influence what is spoken, how it is spoken, and when participants choose to remain silent (see Figure 1 below).

### 2.2 Data collection processes

All focus group discussions were conducted in person in environments familiar and comfortable to the participants. These were in local cafés, bars or fans' official gathering points. The previously mentioned urban segregation was also felt in the venues with their locations being in either predominantly Albanian or predominantly Macedonian neighborhoods in Skopje. Each discussion lasted between 60 and 90 min. To accommodate participants' linguistic preferences, discussions were held in either Macedonian or Albanian language, depending on the ethnic composition of the group. The focus groups took place over a three-week period, and were led by one moderator, the first author of this paper, who is fluent in both languages.

The discussions were audio recorded with the participants' (verbal) consent. Four of the six groups consented to audio recording, while two groups declined to be recorded. In those cases, participants allowed time during the session for the moderator to transcribe responses manually on the spot, while maintaining the flow of the discussion. Verbal informed consent was obtained from all participants at the beginning of each session, following an explanation of the study's aims, procedures, and ethical considerations^2^. All recordings were later transcribed verbatim, anonymized for analysis, and later translated to English. The transcripts were then analyzed using thematic analysis following Braun and Clarke's (49) model, beginning with familiarization and moving through theme development and conceptualization. Themes were interpreted in relation to the study's theoretical framework, with particular attention to expressions of social identity, ethnic boundary, prejudice, and the dynamics of intergroup contact.

### 2.3 Focus group guide and thematic structure of questions

The focus group discussions were guided by a set of questions which derived from the existing literature on the core concepts and theories used in this paper. The questions remained open-ended and flexible, allowing participants to express their experiences in their own terms and enabling new insights and concepts to emerge. Before the exploratory questions, to break the ice and ease participants into the discussion, each focus group began with a brief introduction in which participants were invited to share their age and how long they had been fans. Table 2 outlines the thematic structure of the questions.

**Table 2:** Focus group discussion guide.

Conceptual theme	Questions
Fan identity and group belonging	1. How did you become a fan, and why do you support your team? 2. What makes your fan club unique? What do you appreciate most about being part of this team?
Representation and inclusion in sports	3. Do you know if there are players from different ethnicities in the national team? What do you think about these players? 4. Who can be a national team player? What do you think about the selection process?
Intergroup contact	5. Do you think basketball creates opportunities for better understanding between different communities?
Symbolic expression and intergroup dynamics	6. How do you plan or select songs and slogans for games? Do these reflect your community's identity? 7. Do you collaborate with other fan groups? Any rivalries? Any collaborations? 8. Have you ever interacted with fans from other ethnicities? How was the experience? 9. How does the atmosphere at games reflect relationships between different ethnic communities? 10. What do you think about the North-Macedonian Basketball Federation's logo?

## 3 Results

The results illustrated in this section are organized thematically, based on recurring patterns identified during the thematic analysis. Each sub-section highlights a key theme that emerged across the focus group discussions, supported with participant quotes.

### 3.1 Becoming a fan: “epitome of existence”

Responses around becoming a fan emerged as deeply emotional and identity-forming moments in all six focus groups. The early connection to a team was not described as a casual choice, but as an existential transformation—a moment that gave meaning and structure to the fans' everyday lives:

“I was bored, my cousin invited me to a match and then to a gathering. It left an impression. I read on forums about what it means to be a fan—you build character, you lose the feeling of fear. You gain strength and confidence. It's like something sacred—an indescribable feeling. You can only experience it. It's an escape from the real world. A pressure release. A sense of belonging.”—(FG1 Macedonian fan)

Similarly, a participant from FG2, emphasized the moment of complete merging between the self and the group:

“Started at the age of 13–14, grew out of neighborhood loyalty and poverty, friendship, fighting culture, building something meaningful out of nothing. If the group doesn't exist, we don't exist. I joined once and I never want to leave, for that to happen I'd have to die.”—(FG2 Macedonian fan)

The same intensity was felt among the Albanian fans. For instance, participants framed their identification with the team as something inherited and existentially inevitable:

“You are born a fan, you do not become one. The emotions I feel for this team are out of this world, I can't put them into words. You have to be there to understand it.”—(FG5 Albanian fan)

“The team I cheer for is the icon of our neighborhood, from the moment I found myself among the crowd I just knew it—this is where I belong, it was a moment of rebirth.”—(FG6 Albanian fan)

In the last statement the fan identity is described not as a choice, but as something inherited, as one's ethnic and religious identity may be. In addition, when the focus group participants were asked to describe the demography of their fan groups, they emphasized that these groups are “ethnically pure”.

This parallels Cleland's (1) observation that fan identities in multi-ethnic societies are often deeply entangled with other social identities (e.g., ethnic, religious, cultural), showing that fandom is almost never a leisure activity. Using the word pure further underlines the exclusionary nature of such supporter communities.

These testimonials reflect how fan identity among both ethnic-Macedonian and ethnic-Albanian fans in North Macedonia is connected to personal history, social ties, neighborhood culture, and ethnic belonging. While the reasons behind their entrance to the fan group may vary, in the end they showed total identification with the group.

#### 3.1.1 Style matters

Besides their emotional ties towards one's fan group, the focus group participants also put an emphasis on the symbolic and functional features of their clothing, accessories, and other visual markers of group membership. Far beyond simple aesthetics, style was portrayed as a means for expressing identity, loyalty, belonging and ensuring in-group cohesion.

“We wear dark colors—it indicates maturity, seriousness, and manliness. We have specially designed hoodies which can cover our heads and faces during fights.”—(FG1 Macedonian fan).

This statement illustrates the dual role of fan apparel: symbolic and practical. The choice of dark colors was linked by the participants to values such as seriousness, aggression, and control, qualities often associated with hegemonic masculinity and group strength in sporting subcultures (50). In various youth, fan, and protest cultures, black or dark attire has been used to convey anonymity, resistance, and collective power (50). At the same time, the hoodies serve a practical function during potential fights with other fan groups, suggesting that clothing is as much about performance and preparedness as it is about group identification and solidarity.

The exclusivity and regulation of group merchandise further strengthens this process of internal differentiation:

“The merchandise which we (the fan group members) wear are not for sale. They are printed in a limited number. The leaders of the sub-groups around the neighborhood make lists and order the exact number of t-shirts … We have to be careful to whom we give these merchandise to, only the real fans can wear them.”—(FG2 Macedonian fan)

According to this statement, when it comes to the fan group clothing (and merchandise in general), not everyone is allowed to wear it, it must be earned. This gatekeeping mechanism ensures authenticity within the group and prevents outsiders from presenting themselves as genuine members without having gone through the expected processes of commitment.

“Originality is crucial. From our hairstyle, all the way to our shoes. For example, it is important to wear shoes that do not have loose laces. What if I trip over and fall while I run away from the police?”—(FG5 Albanian fan)

Not only do the clothes complete the fan identity, but they must also meet functional demands such as being able to run, fight, or evade. The concern with originality also speaks to intra-group competition and a desire to distinguish one's own group (or sub-group) from others, reinforcing in-group cohesion and out-group distinctiveness, which are also key aspects of Social Identity Theory.

One of the strongest symbolic functions of fan apparel lies in its reputational weight. As one participant from FG3 explained:

“Songs, clothes and symbols are directly connected to our identity … it tells what kind of character we have.”—(FG3 Macedonian fan).

“The biggest failure of a fan is if their (fan club) scarf, flag or t-shirt is taken away from him during a fight. It is like taking someone's pride if you steal their shirt or scarf—it's an extreme shame if they strip you, some people quit if they lose their shirt.”—(FG3 Macedonian fan).

Here, fan merchandise is treated as something sacred. Losing a scarf was described as losing identity, and in some cases losing the right to further participate due to immense shame. Through clothing and other types of merchandise, fans construct narratives about who they are and who they are not. It is perceived as fundamental to how identity, status and boundaries are managed within the North Macedonian fan culture.

### 3.2 Intergroup bias

#### 3.2.1 Rivalry based on ethnic grounds

Expressions of rivalry between fan groups in North Macedonia are frequently shaped by broader ethnic and identity-based dynamics. The topic of ethnicity was not directly brought to the participants by the moderator, but fans were given the space to bring up this topic if they were willing to. Ethnic-Albanians and ethnic-Macedonians were mentioned spontaneously in all discussions after participants were asked to list fan groups with whom they have the biggest rivalry.

“They (Albanians) are just there to go against us (Macedonians), they are meaningless people. Supposedly the police don't let us attend their matches, but whatever, they are afraid. They want to take over and claim the city. That club is not from this city.”—FG2

In this statement, the out-group is framed as oppositional and external, suggesting a form of boundary where the legitimacy of the other's presence, both in sports and in the urban environment, is questioned. Moreover, the out-group is portrayed as a threat, reflecting not only prejudice but also competition over the symbolic ownership of space.

“It's not bad to be a nationalist. I love what's mine, but if someone doesn't love what's mine, I won't sit quietly.”—(FG3 Macedonian fan).

This quote expresses a defensive form of nationalism, where love for one's group and symbols is seen as valid, but any perceived disrespect from the out-group justifies retaliation. Such attitudes further support the idea that prejudice is not only rooted in identity but also in the belief that one's group is under threat. As RCT suggests, this perceived threat fosters intergroup hostility and entrenches division (15).

From the perspective of Albanian fans while speaking of their rivalry with ethnic-Macedonian fans, the narratives included descriptions of identity concealment and restricted access to games, suggesting the existence of perceived or actual barriers (such as entering a game):

“Sometimes I put on clothes so I look like a Macedonian, this way I can hide my identity.”- (FG4 Albanian fan).

“In some (critical) games they do not allow us to enter due to previous incidents … At the ticket office they check our IDs, if it is an Albanian name/surname, they do not sell it to us.”—(FG4 Albanian fan).

“Some of us found Macedonian friends and asked them to buy tickets for us.”—(FG5 Albanian fan).

These insights contribute to better understanding of how fans reveal that sports becomes not just a stage for (verbal) expression but a symbolic battleground in multiethnic contexts.

#### 3.2.2 Contested symbols and national identity

To understand the role of national representation in sports, the focus groups included a visual prompt—the logo of the Basketball Federation of North Macedonia, which underwent a minor (visual) change (see Figure 2) where the word “North” and the letter “N” were added after the change of the country's name.

The reactions towards the logo were highly emotional, revealing how logos and state branding elements are interpreted through the lenses of national identity, ethnic belonging, and resistance:

“It looks like a helicopter. Our flag is the Vergina Sun. I don't agree with the letter ‘S’—we are not ‘North’.”—(FG1 Macedonian fan).

“I don't like the ‘North’ in the logo—it reflects politics which goes against who we really are and we are not the North.”—(FG2 Macedonian fan).

These statements indicated a strong rejection of the term “North”, especially among Macedonian participants, who saw it as a threat to their national sovereignty and identity. The letter “S” (Severna in Macedonian, meaning North) in the Macedonian version of the logo caught the attention of the participants within the first few seconds after the moderator raised the image towards the group. In several discussions, the Vergina Sun was invoked as the “true” national symbol, reflecting a deeper desire for ethnic and historical authenticity, and indicating the dissatisfaction with the 2019 Prespa Agreement, which changed the country's name and national symbolism. The mention of banned flags in sporting events further reinforced the feeling of suppression and marginalization of what fans viewed as rightful heritage. Keeping old ID cards (which was the case with many of the participants) or refusing to acknowledge the name change became symbolic acts of protest. These expressions indicated where resistance is framed as loyalty and patriotism, and institutional shifts in national representation are seen as betrayals.

“Dislike for the ‘S’ (as in ‘Severna’), I also refuse to change my personal ID, and feel as if my identity and history are erased with the word ‘North’.”—(FG3 Macedonian fan).

Many of the participants in the focus group consisting of ethnic-Macedonians shared that they had decided to keep their old IDs as a sign of resistance and protection of their history, legacy, and identity.

For ethnic-Albanians, the criticism for the logo was present, but framed differently:

“I find the logo of the Federation the same as the logo of McDonalds, it's hilarious.”—(FG5 Albanian fan).

The responses among ethnic-Albanian fans suggested detachment and alienation rather than betrayal or threat. The participants did not react to the additional word “North”, neither felt the need to further continue to comment on the logo. While some mocked the logo's aesthetic—likening it to a fast-food brand, for example the addition of “North” did not provoke a sense of betrayal or cultural loss. This suggests that for many ethnic Albanian fans, the name “North Macedonia” carries far less affective weight. Rather than seeing the name change as a threat to their identity, some Albanian participants expressed skepticism toward state symbols in general, viewing them as imposed, unrepresentative, or simply irrelevant. Their silence or disengagement from the logo debate might be understood not as apathy but as a commentary on exclusion. In other words, when one does not feel fully represented by the state, one is less likely to contest or defend its symbolic changes.

In view of the findings among ethnic Macedonian fans, these design elements were experienced as symbolic threats to group identity, national continuity, and personal history, rather than being perceived as neutral administrative language. While previous literature has highlighted the role of sports in expressing nationalism, primarily through chants, rivalries, and fan violence (1, 12), this study has uncovered a new layer by revealing how institutional design choices, such as federation logos, are actively contested by fans. However, while ethnic-Macedonian fans were deeply affected by this change in the logo, the reactions of ethnic-Albanian supporters rather reflected their detachment from the country's national symbols and political developments.

### 3.3 Extended contact among fans

#### 3.3.1 Who can be part of the (national) team?

To explore whether the ECH is relevant and to better understand fans' attitudes towards ethnic out-group members within the teams they support, participants across all focus groups were asked to reflect on who qualifies to play for the (North Macedonian) national team and who are the preferred types of players to have in the clubs they support. While most participants accepted the idea that players with different ethnic backgrounds could play for their team or even the national team, this inclusion was always conditional:

“As long as they contribute, they can play. They've eaten and drunk from this country; we raised them here—they can be given a chance. But they can also play for Albania—give them a passport, let them play there.”—(FG1 Macedonian fan).

“If he gives his best for the country and puts it beyond his ethnicity, he can play.”—(FG2 Macedonian fan).

The statements reflect a clear boundary on belonging where the inclusion of ethnic-Albanians in national teams, or the local teams, is acceptable only if they meet specific criteria and performance. Even though current ethnic-Albanian players are part of the national teams (in multiple sports) and are North Macedonian citizens with equal status, they are not perceived as equals. Neither did the shared group identity among the players, which is one of core requirements for successful ECH, show a positive impact regarding the attitudes towards out-group members among fans.

Ethnic-Albanian fans had the same perception of conditional acceptance, even in cases where ethnic-Macedonian players had contributed positively to their team in the past few years:

“We have players of many ethnic and national groups, there are Macedonians as well. We like the Macedonians who play for us but I would prefer not to be touched by them. For example, at the end of the game if they come to shake our hands, we deny it. As long as they bring us the championship, we do not care.”—(FG6 Albanian fan).

Here, the Macedonian player is accepted as a tool for winning, but still physically and symbolically excluded. The denial of a handshake, a small gesture of human reciprocity, further shows the failure of affective contact. With this, we can see that the presence of an out-group member is not enough to reduce prejudice when emotional and symbolic barriers remain intact.

When it comes to the ethnic-Albanian players representing North Macedonia in various European Championships, the participants shared their disappointment in the players' acceptance to participate and be part of the national team:

“For the talented (ethnic) Albanian players, if they want to play for the national team, they can simply buy the passport and play for Albania. They have the option. So, if they play for Macedonia, it is their choice, which is not the best.”—(FG5 Albanian fan).

Rather than reflecting successful intergroup cooperation, this view reaffirms that national teams are still seen as ethnically owned entities, where cross-ethnic participation is a betrayal or at least a morally compromised choice. Additional positive experiences shared by ethnic-Macedonian fans were dismissed because they did not meaningfully challenge their broader social outlook:

“We also had Albanian players in our team, but this doesn't change our reality.”—FG3

These responses show that contact among the players of different ethnic groups is interpreted by the fans through the lens of instrumentality rather than shared identity. In the absence of affective investment, reciprocal recognition, and structural support, these moments of contact fail to generate broader shift in intergroup attitudes among the fans.

While players with different ethnic and religious backgrounds may bond over their shared athletic goals, the lived experience of fans is rarely mutual. What is experienced as a symbolic inclusion by one group may at the same time be interpreted as a symbolic loss or erosion by the other, highlighting how affective tensions are not just personal but politically charged and collectively reinforced.

#### 3.3.2 Conditional inclusion and symbolic distance: “We will come together in hell”

One of the central promises of sport in multi-ethnic societies is its potential to serve as a platform for social dialogue, cohesion and inclusion, offering shared experiences that go beyond ethnic, religious, or political differences. However, the data from all six focus groups reveal a more complex and rather pessimistic picture in the case of North Macedonia. Instead of functioning as a unifying arena, we found that sports can often serve as an extension of ethnic divisions, political interests, and distrust.

The participants were asked whether they find sports as a potential platform for inclusion of diverse groups, including fan groups with different ethnic backgrounds:

“No, sport is corrupted by sponsors and money. It's a breeding ground for nationalists.”—(FG2 Macedonian fan).

This statement reflects on the integrity of sports institutions, suggesting that what might have once been an arena for unity has become hijacked by private and public interests.

The following quotes voice a more structural critique, pointing to how political elites and institutions may actively resist or block integrative potential.

“Sports can be used as a platform for inclusion … but sometimes it goes against the interest of certain entities and institutions in this country.”—(FG5 Albanian fan).

“Sometimes sports are being abused for nationalistic interests.”—(FG6 Albanian fan).

Rather than fostering inclusion, sports are viewed as instrumentalized spaces to exploit nationalist rhetoric or political gain. One of the participants gave a more context-specific example of why sports may not work as a tool for inclusion:

“Sport can unite people, but not with Albanians—they don't love this country.”—(FG3 Macedonian fan).

Here, the possibility of inclusion is explicitly ethnically bounded. While sports may unite “us” (in-group members), that unity is denied to “them” (ethnic-Albanians), who are viewed as disloyal or even hostile to the nation. This attitude is a clear example of in-group favoritism and out-group derogation (19), where inclusion is conditional upon perceived loyalty to national identity.

Ethnic-Albanian fans also shared similar resistance when it came to using sports as a platform for coming together:

“Nothing can bring us together. We are like oil and water. We simply do not mix. We will come together in hell, end of story.”—(FG4 Albanian fan).

The metaphor of “oil and water” speaks not only to difference, but to incompatibility, and the idea of “coming together in hell” represents the depth of resentment that overcomes any imagined sportsmanship or unity.

### 3.4 Ambivalence and contradictions in fan discourse

While the previous sub-sections of the analysis highlighted clearer divisions between in-group and out-group attitudes, this final section looks into the tensions and inconsistencies that emerged within the members of the fan groups during the focus group discussions. There were moments that revealed emotional and ideological contradictions in how fans spoke about belonging, loyalty, and identity. These ambiguities help us understand prejudice beyond ideology, as a practice used among the participants to belong, survive, and maintain relationships.

For instance, across nearly all focus groups, humor appeared as a powerful tool for both regulating and reinforcing belonging. Fans mocked each other using the same language they used against rival groups.

“He dresses just like them.”—(FG2 Macedonian fan)

“That haircut? He's looks like he is from the other side of the bridge.”—(FG2 Macedonian fan)

They also often teased each other about being uneducated even while calling rival fans illiterate.

“You? You can't even read, just like the others from the other part of the city.”—(FG4 Albanian fan)

Although no women were included as participants in the focus groups, gendered discourses emerged in how male fans spoke about women who would like to be part of the fan group and in their behavior toward the female moderator. The moderator, as a female researcher, was treated with hospitality by being offered drinks, sweets, and polite attention. Yet within the same conversations, participants spoke about women as outsiders to fan culture, naming them as not “real” fans, but individuals who exploit the group for personal gain, such as access to tickets or status.

“They're not real fans, they come to take photos, to get free tickets and just hang out.”—(FG5 Albanian fan)

This dual positioning, personal respect vs. group-level exclusion, which was also seen towards players of different ethnic groups, reveals how emotional warmth can cohabit with structural marginalization. Prejudice is not always aggressive or explicit, it can be couched in moral arguments and affective gestures that ultimately preserve inequality.

Fans also described the group as a space of complete freedom, a place where they could shout, move, and feel without restraint. Yet, this sense of freedom existed within strict hierarchies, for instance only certain members were permitted to wear specific merchandise, lead chants, or make decisions.

“You can't just wear the group hoodie. You have to earn it. You have to prove yourself.”—(FG1 Macedonian fan)

These practices point toward a form of internal boundary-making that mirrors the very structures fans claim to oppose.

Tensions were also visible in how fans spoke about space. On the one hand, strong territorial attachment was emphasized, with “our side” being described as morally and culturally superior. On the other hand, several participants admitted that they cross over into the “other side” of the city for better coffee or shopping.

“I go for coffee there, some cafes are better but I make sure to never go alone.”—(FG3 Macedonian fan)

“They have got the best place for sneakers. I just go, get what I need, and leave.”—(FG5 Albanian fan)

These moments reveal that even strong symbolic boundaries are often crossed in practice, and that feelings of loyalty and contradiction coexist, sometimes within a single sentence.

Finally, there were moments in the discussions where participants looked to one another for non-verbal approval before speaking, hesitated when naming out-groups, or softened the tone when expressing prejudice.

“They're … not really like us. But you know, I don't mean it badly.”—(FG6 Albanian fan)

These subtle shifts reflect the pressures of speaking in a group setting and the emotional management required to perform loyalty, toughness, and belonging.

Altogether, these tensions help us move beyond understanding prejudice as a fixed ideology or set of beliefs. Instead, they reveal how prejudice is often shaped by emotional discomfort, moral ambivalence, and social performance. While previous sections showed how identities are marked and defended, this section shows how contradictions are part of that same process, offering a deeper, more complex view of how belonging and exclusion operate in the everyday experiences of fans.

## 4 Discussion

Our study explored how ethnic identity and intergroup prejudice are expressed in the basketball fandom in North Macedonia, offering a perspective on the dual role of sport in multi-ethnic and post-conflict societies. While international organizations and sport-for-development advocates often promote sport as a tool for inclusion and peacebuilding, our findings show that fan culture can reproduce and even reinforce social divisions through the same tool.

According to our focus group discussions with basketball fans coming from ethnic-Macedonian and ethnic-Albanian communities, fan identities were shown to be closely tied to ethnic belonging and expressed through symbolic practices such as clothing, chants, rituals, and slogans. These boundary-marking attitudes reflect the dynamics described through the Social Identity Theory (19), where group membership becomes a key source of self-definition and intergroup differentiation as well. This kind of “hot” fandom can also be connected to Giulianotti's (12) typology, which highlights supporters' emotional loyalty and local affiliation, strengthening in-group relations and out-group exclusion. In this context, supporting a basketball team becomes more than a leisure activity, it turns into a performance of belonging and a statement of ethnic and territorial identity.

This depth of emotional investment is especially evident in how fans describe their connection to the team. As shown through the lens of Jacobson's (8) fan identity development framework, this connection moves from initial attraction to identity internalization, where fanhood becomes a deep expression of self. Fans did not present their identities as performative or casual but rather as emotionally intense, rooted in sacrifice, loyalty, and territorial attachment. This was further underlined by the use of terms like “sacred,” which were not metaphorical, but rather pointed to a lived experience of devotion and suffering for the group. These descriptions reveal how fan identity functions as a form of moral belonging and social commitment, often sustained through hardship, neighborhood ties, and perceived group responsibilities.

Similarly, practices such as wearing group-specific clothing and merchandise reflect the principles of both Social Identity Theory and enclothed cognition. Uniformity in dress not only marks symbolic boundaries between groups but also enhances internal group cohesion and identity. Giulianotti's (12) concept of the “supporter” identity type aligns with these practices, highlighting long-term emotional investment and moral economies of loyalty where items like t-shirts and scarves are not just accessories but symbols of recognition and responsibility.

As a result, the expression of prejudice according to our focus group participants' lived experiences went beyond verbal hostility. Participants described being exposed to exclusionary strategies, such as their surnames being checked by the ticket office staff in the gyms, suggesting that ethnic bias is also institutionalized and routinized. These narratives reflect deeper perception of competition beyond the games, done over symbolic and physical space, aligning with Realistic Conflict Theory (14, 15), which explains how perceptions of zero-sum competition can lead to sustained intergroup hostility. As an outcome, the context of fandom turns into a symbolic battlefield, where group status, space, and legitimacy are negotiated. And, while the simple existence of the outgroup is perceived as problematic, it also provides a reference point that is vital for in-group identification.

In this sense, the city itself becomes a contested space, “claiming the city” emerges not just as a spatial reference but a symbolic and political act. Realistic Conflict Theory is particularly useful here, as the fans' territorial attachments mirror intergroup competition over symbolic resources such as identity, status, and legitimacy. Social Identity Theory complements this by highlighting how identity is actively reinforced through contrast with a perceived out-group, especially in shared or divided urban environments.

Although the Extended Contact Hypothesis (20) offers a promising framework for prejudice reduction, our findings indicate that its effectiveness is limited in highly segregated and polarized societies, such as the one in North Macedonia. The acceptance of out-group players (in this case, ethnic-Albanian players), was highly conditional, often based on assimilation, performance or loyalty. It also rarely translated into broader positive attitudes towards the entire (ethnic) group. While players with different ethnic or religious backgrounds can be brought together under one team having a common goal and the shared experience of their athletic identity, the experience of fans is seldom shared, what is perceived as a symbolic gain in one group can be seen as a loss in the other—particularly when ethnic identities are tied to perceived group status, territorial belonging, or political legitimacy.

To further understand this dynamic, affect theory offers a valuable lens: emotional responses are not merely personal but are shaped through social narratives, collective histories, and political hierarchies. Inclusion without emotional resonance remains hollow. While fans may cognitively accept the presence of an out-group player, emotional attachments such as trust, empathy and respect do not automatically follow. As such, the transformative potential of contact—even when present—is often limited by emotional distance, unprocessed historical grievances, and symbolic boundaries that remain intact.

This limitation is further reflected in the broader socio-political landscape. As Doidge (2) argues, fan cultures can mirror political divisions, with institutional interference and sponsorship dynamics shaping how contact occurs. Even when individuals within fan groups express openness, external factors such as political agendas or ethnic polarization can prevent this openness from translating into real attitudinal change.

This study also has several limitations. The sample consisted only of male fans, which reflects the gender imbalance in organized basketball fandom in North Macedonia but limits our understanding of how gender intersects with ethnic identity and fan culture. Additionally, important themes such as the influence of political parties, urban space division, and the use of hate speech in online fan spaces were raised during discussions but could not be included in the analysis.

Based on our findings, we offer several recommendations. First, interventions aiming to reduce prejudice through sport must go beyond symbolic gestures, and besides initiatives for connecting athletes, it is crucially important to promote meaningful interaction between fans of different ethnic backgrounds.

Although the presence of ethnically mixed teams was tolerated by fans, our data show that this form of extended contact often failed to lead to deeper attitudinal change. Therefore, beyond integrating athletes, it is crucial to promote meaningful, emotionally resonant interaction between fans of different ethnic backgrounds. This could take the form of joint supporter initiatives, community tournaments, or fan-led dialogue sessions facilitated by trusted figures.

Second, the study revealed structural barriers that fans from minority backgrounds face, such as exclusion from matches based on names or perceived ethnicity, and alienation from national symbols like the federation's logo. Therefore, institutions such as sports federations, clubs, and municipalities should take a more active role in designing inclusive policies, ensuring equitable access to games, enforcing anti-discrimination protocols, and creating mechanisms to report and respond to bias.

Third, our findings showed that younger fans and fan sub-groups often absorb and reproduce existing prejudices, and to counter this issue we recommend integrating educational campaigns targeting younger fans and fan sub-groups into the educational system, existing club infrastructures, using former players to promote narratives of inclusion, mutual respect, and shared loyalty to the team.

Finally, our participants expressed deep emotional attachment to identity, tradition, and belonging, which shaped their perceptions of legitimacy, rivalry, and territorial ownership. Hence, the interventions must be sensitive to the emotional and symbolic weight fans attach to these matters. Change is unlikely to emerge from top-down policies alone; it must also be cultivated from within fan communities. These findings contribute to a more nuanced understanding of contact-based interventions in divided societies. While CBIs often rely on structured opportunities for contact—such as shared teams, joint activities, or co-fan events—our study shows that without addressing emotional resonance, symbolic boundaries, and underlying narratives of belonging and exclusion, such interventions may remain superficial. Emotional detachment, conditional acceptance, and symbolic resistance emerged as barriers that limited the impact of extended contact. Therefore, effective CBI in contexts like North Macedonia must go beyond physical proximity and include emotionally meaningful encounters that acknowledge the political and symbolic weight of identity and rivalry. Collaborating with fans, not against them, will be essential for reimagining sport as a space where rivalry does not turn into disrespect, and where identity can be expressed without exclusion.

## Data Availability

The original contributions presented in the study are included in the article/Supplementary Material, further inquiries can be directed to the corresponding author.

## Footnotes

^1^While data on gender composition of the basketball fan groups in North Macedonia is not available, according to the fan group leaders who were part of the focus groups and based on observations from fan group events and games, female participation is minimal. In addition, leadership positions within these fan groups are all held by men.

^2^This research received ethical approval from [anonymized institution].
